# Carrot Genotypes Contrasted by Root Color and Grown under Different Conditions Displayed Differential Pharmacological Profiles in Vascular and Metabolic Cells

**DOI:** 10.3390/nu12020337

**Published:** 2020-01-27

**Authors:** Raffaella Soleti, Patricia Mallegol, Grégory Hilairet, Mehdi Frifra, Florent Perrin, Cécile Dubois-Laurent, Sébastien Huet, Pascale Pignon, Laetitia Basset, Emmanuel Geoffriau, Ramaroson Andriantsitohaina

**Affiliations:** 1 SOPAM, U1063, INSERM, UNIV Angers, SFR ICAT, 49100 Angers, France; patricia.mallegol@univ-angers.fr (P.M.); gregory.hilairet@univ-angers.fr (G.H.); mehdigf@hotmail.fr (M.F.); ramaroson.andriantsitohaina@univ-angers.fr (R.A.); 2 IRHS, Agrocampus Ouest, Inrae, Université d’Angers, SFR Quasav, 49045 Angers, France; fp.florentperrin@gmail.com (F.P.); cecile.dubois@agrocampus-ouest.fr (C.D.-L.); sebastien.huet@agrocampus-ouest.fr (S.H.); emmanuel.geoffriau@agrocampus-ouest.fr (E.G.); 3 CRCINA, INSERM, Université de Nantes, Université d’Angers, 49100 Angers, France; pascale.pignon@univ-nantes.fr (P.P.); laetitia.basset@univ-angers.fr (L.B.)

**Keywords:** carrot extracts, vascular cells, metabolic cells, oxidative stress, apoptosis, proliferation, lipid accumulation, inflammation

## Abstract

Carrots’ genotype and growing conditions influence their potential properties to fight against cardiovascular and metabolic diseases. The present study evaluated the influence of carrot genotypes contrasted by root color (Bolero, Presto, Karotan, Deep Purple, Kintoki and Blanche des Vosges) growing under standard, water-restricted, biotic stress (*Alternaria dauci* inoculation), and combined stress conditions (water restriction and *A. dauci* inoculation). The effect of carrots’ polyphenol and carotenoid content was assessed on endothelial and smooth muscle cells, hepatocytes, adipocytes and macrophages functions (oxidative stress, apoptosis, proliferation, lipid accumulation and inflammation). Independently of varieties or growing conditions, all carrot extracts affected vascular cells’ oxidative stress and apoptosis, and metabolic cells’ oxidative stress and lipid accumulation. Three clusters were revealed and displayed beneficial properties mostly for adipocytes function, smooth muscle cells and hepatocytes, and endothelial cells and hepatocytes, respectively. Karotan and Presto varieties exhibited endothelial tropism while Blanche des Vosges targeted adipocytes. Carrots under biotic stress are more efficient in inducing beneficial effects, with the Bolero variety being the most effective. However, extracts from carrots which grew under combined stress conditions had limited beneficial effects. This report underscores the use of certain carrot extracts as potential effective nutraceutical supplements for metabolic diseases.

## 1. Introduction

Metabolic disorders including obesity, glucose intolerance, high blood pressure and dyslipidemia are associated with increased risk of type 2 diabetes and cardiovascular diseases which are known to be oxidative stress-related [1]. The management of these interconnected factors is complex and natural products and plant-derived foods are widely recommended for their health-promoting effects [2,3,4] typically associated to their antioxidant, phytoestrogen and anti-inflammatory properties.

Carrot root has relevant interest in preventive nutrition due to its richness in bioactive compounds including carotenoids, vitamins, polyphenols, fiber and minerals [5,6]. The abundance and diversity of those bioactive compounds may explain the health effects associated with carrot consumption. Indeed, in cholesterol-fed mice, carrot ingestion reduces plasma and hepatic lipids and improves antioxidant status and risk factor for cardio-vascular diseases especially in nutrition-induced stress conditions [6]. In a rat model of metabolic syndrome, carrot juice attenuates or reverses the changes in cardiovascular and liver structure and functions as well as in metabolic parameters, especially abdominal fat deposition and plasma lipid profiles [7] with its lipid-lowering effect of carrot mainly mediated its fiber component [6]. Oral administration of carrot extract also induces a significant protective action in the alleviation of hepatocellular injury in a mouse model of acute liver damage [8]. It also enhances antioxidant defenses which could be explained by the high carotenoid and fiber content of this vegetable [9]. Indeed, carrot antioxidants including carotenoids and polyphenols are able to quench free radicals and enhance endogenous systems of defense. These properties concur to the reduction of oxidative stress and the subsequent decrease of the associated risk of degenerative and cardiovascular diseases [10,11,12]. The elevated concentration of carotenoids, mostly β-carotene, in carrot roots has immune enhancers, anti-mutagenic and anti-cancer properties, and dietary carrot intake is associated with decreased risk of breast cancer [13] and is inversely associated with prostate cancer risk [14]. Finally, a human study showed that drinking carrot juice may protect the cardiovascular system by increasing total antioxidant status, lipid peroxidation and systolic blood pressure [15].

The composition and content of bioactive compounds of carrot root could be affected by various parameters, including genetic background, geographic location, climate, physiological age and growing conditions. Increasing evidence also have suggested that the health benefits of fruits, vegetables, whole grains and other plant foods can be attributed to the synergy or interactions of bioactive compounds and other nutrients of the whole foods [16].

In the present paper, we studied the effect of those various parameters by analyzing extracts from carrots from Bolero, Presto, Deep Purple, Karotan, Kintoki and Blanche des Vosges varieties grown under (i) standard conditions (C), (ii) water-restricted condition (HR), (iii) biotic stress conditions with *A. dauci* inoculation (BS) and (iv) combined stress conditions with both water restriction and *A. dauci* inoculation (CS).

As previously reported [17], a protocol developed to evaluate the biological functions of cells involved in metabolic diseases such as vascular cells (endothelial and smooth muscle cells), metabolic cells (hepatocytes and adipocytes) and immune cells (macrophages) were used, including the measure of oxidative stress, apoptosis, proliferation, lipid accumulation and inflammation markers. This screening of pharmacological responses at a cellular level will allow the identification of the most promising carrot extracts to be tested further on animal model of metabolic diseases to test the potential use of carrot and/or carrot-derived products in management of metabolic pathologies.

## 2. Materials and Methods

### 2.1. General Strategy

**Figure 1:**
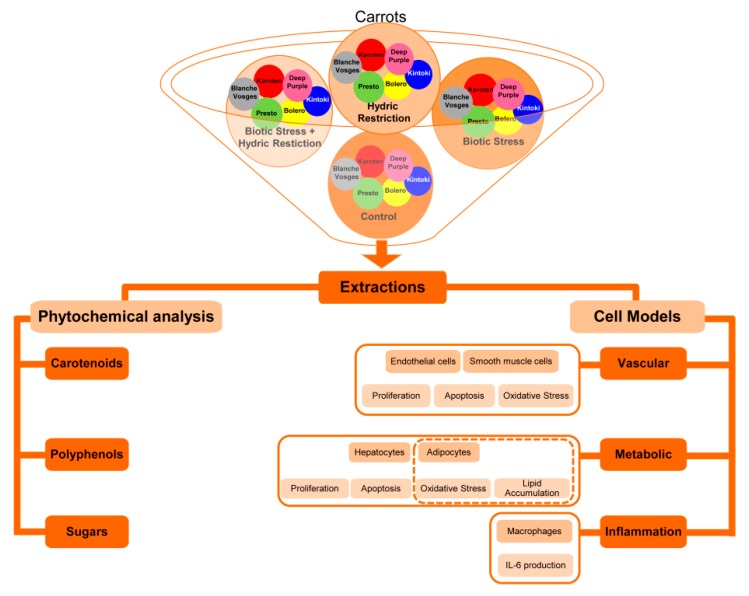
Diagram showing general strategy used during the project. Frozen homogenates from six carrot varieties (Bolero, Presto, Deep Purple, Karotan, Kintoki and Blanche des Vosges) grown in (i) standard conditions (control), (ii) water-restricted condition (hydric stress), (iii) *A. dauci* inoculation (biotic stress), or (iv) water restricted and *A. dauci* inoculation combined conditions (combined stresses) were solubilized in either ethanol or DMSO. Each extracts were tested at two different concentrations on the function of vascular (endothelial and smooth muscle cells), metabolic (hepatocytes and adipocytes), and immune (macrophages) cells. The ability of extracts to counteract oxidative stress in vascular and metabolic cell models was assessed; as well as their capacity to prevent apoptosis and to modulate proliferation of endothelial, smooth muscle cells and hepatocytes. Extract ability to modify lipid accumulation in hepatocytes and adipocytes as well as pro-inflammatory cytokine IL-6 in macrophages was evaluated. In parallel the phytochemical analysis of carotenoid, polyphenol and sugar content of extracts was performed.

Frozen carrot homogenates from six different carrot varieties whose genotypes were contrasted by their root color were solubilized in either ethanol or DMSO. Simultaneously, each extract was tested at two different concentrations on the cellular models described previously (Figure 1) and a phytochemical analysis of carotenoid and polyphenol content was carried out.

### 2.2. Plant Material and Sampling

Six different carrot genotypes contrasted by their root color have been studied: three varieties, Bolero (orange), Presto (orange) and Deep Purple (purple); and three inbred lines derived from Karotan (orange), Kintoki (red) and Blanche des Vosges (white). Plants were grown under a semi-controlled environment in tunnel in two biological repetitions in 2013 at Agrocampus-Ouest (IRHS-Angers, France). Plants were grown in (i) standard growing conditions (no inoculation, soil potential 31.6 kPa, control, C), (ii) water-restricted condition (soil potential 316 kPa, HR), (iii) biotic stress conditions with *A. dauci* inoculation (BS), and (iv) combined stress conditions with both water restriction and *A. dauci* inoculation (CS), as described by Perrin et al. [18]. *A. dauci* artificial inoculation was done using a solution of 4000 conidia/mL. The first inoculation was performed when plant showed three true leaves and the second one 15 days later. The inoculation part of tunnel was separated from the healthy one by a waterproof tarp [18]. At harvest, 10 plants per genotype and replication were collected. Three medial parts of the root were taken from each root, grinded in bulk and immediately stored at −80 °C.

### 2.3. Carrot Extract Preparation

Twenty milligrams of frozen carrot from both biological replicates (1:1) were solubilized in 2 mL of two polar solvents (100% ethanol [EtOH] or dimethysulfoxide [DMSO]) and vortexed during 10 min at room temperature protected from light. Then, the solutions were filtered and freshly used at 10^−2^ (high concentration, H) or 10^−5^ g/L (low concentration, L). We have taken into account the combination of the responses obtained with the two solvents to gain the most information and maximize the mode of extraction. Ethanol and DMSO were used at concentrations (less than 0.1%.) at which no cytotoxicity was observed, as deduced from trypan blue exclusion.

### 2.4. Determination of Carotenoid Content in Carrot Extracts

The carotenoid composition was analyzed in the ethanol extracts only, as DMSO is a solvent methodologically incompatible with system used in our experimental condition. Carotenoid content in 100µL of ethanol extracts was measured according to Perrin et al. [19] by HPLC system (Shimadzu Corporation, Kyoto, Japan) equiped with a thermostated autosampler (SIL-10AD VP), a diode array detector (SPD-M10A VP) and aYMC C30 (YMC, Japan) column (150 × 4.6; 3 μm). The elution program was as followed for solvents A and B: 0–12 min, 80–45%; 12–14 min, 45–10%; 14–17 min, 10–80%; and 17–23 min, 80%. Carotenoid contents were expressed as µg/mL of equivalent β-carotene.

### 2.5. Determination of Phenolic Content in Carrot Extracts

Total polyphenol content in extracts was measured by Folin-Ciocalteu reagent according to Everette et al. [19]. In brief, 100 µL of ethanol extract or 200 µL of DMSO extract was mixed with 100 µL of Folin-Ciocalteu reagent and 1.58 mL, homogenized and incubated during 5 min. Then 0.3 mL of Na_2_CO_3_ (20% *w*/*v* in water) was added followed by an incubation 30 min at 45 °C before measuring the absorbance at 765nm. The control was made by solvent reaction alone. Results were expressed in µg/mL equivalent gallic acid. This phenolic content assay can be seen as a measure of the total antioxidant capacity [20].

### 2.6. Cell Culture

As previously described [17], pooled primary human umbilical vein endothelial cells (HUVECs) were obtained from Lonza and cultured in EGM-2 endothelial medium BulletKit system (Lonza, Basel, Switzerland) consisting of endothelial basal medium supplemented with 5% fetal bovine serum (FBS), vascular endothelial growth factor (VEGF), fibroblast growth factor-2 (FGF-2), epidermal growth factor (EGF), insulin growth factor-1 (IGF-1), ascorbic acid, gentamicin sulfate amphotericin, hydrocortisone and heparin and maintained at 37 °C and 5% CO_2_. HUVECs were used between passages 2 and 6.

Human aortic smooth muscle cells (HASMC) were obtained from Gibco and cultured in medium 231 (Gibco) supplemented with smooth muscle growth supplement (SMGS) consisting of FBS, FGF-2, EGF, heparin, recombinant human insulin-like growth factor-I, and BSA (Gibco) at 37 °C and 5% CO_2_. Cells between passages 3 and 6 were used in all experiments.

3T3-L1 pre-adipocytes were maintained in high-glucose Dulbecco’s modified Eagle’s medium (DMEM) with 10% donor calf serum at 37 °C and 10% CO_2_.

HepG2 cell line was obtained from ATCC and cultured in DMEM high-glucose medium (Lonza) supplemented with 5% FBS. Cells were maintained at 37 °C and 5% CO_2_.

Raw 274.6 cell line was obtained from ATCC, cultured at 100 × 10^3^/mL in complete DMEM and maintained at 37 °C and 5% CO_2_.

The concentration of cells used was chosen as the optimal concentration to obtain the maximal signal/noise ratio (i.e., in the absence and the presence of designated stimuli depending on the cell type).

### 2.7. Proliferation Assay

As previously described [17], effects of carrot extracts on proliferation on HUVECs, HASMCs or HepG2 were analyzed by using CyQUANT Cell Proliferation Assay Kit (Molecular Probes, Eugene, OR, USA). Briefly, 10 × 10^3^ HUVECs and 5 × 10^3^ HASMC per well were seeded into 96-well-plates in medium deprived of serum and allowed to attach overnight. Then, cells were treated with 10^−2^ and 10^−5^ g/L of carrot extracts with either Ethanol or DMSO for 1h and then treated or not with VEGF (20 ng/mL) for further 23 h. HepG2 were seeded at concentration of 20 × 10^3^ cell per well into 96-well plates and allowed to attach overnight. Then, they were treated with 10–2 and 10–5 g/L of carrot extracts with either Ethanol or DMSO for 1h and then supplemented or not with 30% FBS for further 23 h. After treatments, growth medium was removed, dye-binding solution was added into each microplate well and cells were incubated at 37 °C for 1 h. The fluorescence levels were read on a fluorescent microplate reader (Mithras LB 940, Berthold technologies, Baden Württemberg, Germany) with filters for ∼480 nm excitation and ∼530 nm emission.

### 2.8. Apoptosis Assay

As previously described [17], evaluation of hypodiploid DNA was determined by flow cytometry. Briefly, HUVECs (10 × 103) or HASMCs (5 × 10^3^) were seeded in 96-well-plates, HepG2 (10 × 10^4^) were seeded in 24 well plates and allowed to attach overnight. Then, cells were treated for 1 h with or without 10^−2^ and 10^−5^ g/L of carrot extracts, followed or not by addition of the pro-apoptotic agent Act D (1 μg/mL) for 23 h. After treatments, culture medium was removed, and adherent cells were trypsinized, combined with floating cells from the original culture medium, and centrifuged. Cells were then fixed in 70% ethanol for at least 4 h at 4 °C and washed in 0.01 M phosphate buffered saline (PBS, NaCl 0.138 M, KCl 0.0027 M, Sigma Aldrich) before resuspension for 10min in a solution containing type I-A RNase A (0.05 mg/mL, Sigma Aldrich) in PBS at 37 °C. Propidium iodide (PI, Sigma Aldrich) was then added at a final concentration of 12.5 μg/mL. After 15 min in the dark at room temperature, samples were analyzed by MACSQuant^®^ Flow Cytometers (Miltenyi Biotec).

### 2.9. 3T3-L1 Differentiation

As previously described [17], 3T3-L1 pre-adipocytes (30 × 10^3^) were seeded in 96-well-plates, they were induced to differentiate two-days post-confluence in classical medium in the presence of 10% FBS, 3-Isobutyl-1-methylxanthine (IBMX, 250 μM), dexamethasone (1.25 μM), insulin (250 nM) in presence or absence of 10^−2^ and 10^−5^ g/L of carrot extracts with either ethanol or DMSO for 3 days. Then, cells were cultured with 100 nM insulin with or without 10^−2^ and 10^−5^ g/L of carrot extracts with either ethanol or DMSO until complete adipocyte differentiation and, consequently, lipid accumulation (day 7, the medium was replaced each 2 days).

### 2.10. Lipid Accumulation in Hepatocytes

As previously described [16,17], HepG2 (30 × 10^3^ per well) were seeded into 96-well-plates and allowed to attach overnight. Then, HepG2 were treated or not with 10^−2^ and 10^−5^ g/L of carrot extracts with either ethanol or DMSO for 30 min and then in the absence or presence of oleic acid at 500 μM for 24 h. Lipid accumulation was quantified by Oil Red O (See below).

### 2.11. Reactive Oxygen Species (ROS) Quantification

As previously described [17], the fluorescent probe dihydroethidium (DHE, Sigma Aldrich, Saint-Quentin-Fallavier, France) was used to measure the intracellular generation of ROS. Briefly, 10 × 10^3^ HUVECs, 5 × 10^3^ HASMC, 30 × 10^3^ HepG2 per well were seeded into 96-well-plates and allowed to attach overnight. Then, cells were treated with 10^−2^ and 10^−5^ g/L of carrot extracts with either ethanol or DMSO for 23 h followed or not by 1h of H_2_O_2_ (1% for HUVECs, 0.1% for HASMCs and HepG2) stimulation at 37 °C. In another set of experiments, upon differentiation protocol, 3T3-L1 cells were treated for 1 h with or without 1% H_2_O_2_. After treatments, cells were incubated in 3 μM DHE for 1h at 37 °C. The fluorescence intensities of DHE were measured by MACSQuant^®^ Flow Cytometers (Miltenyi Biotec).

### 2.12. Oil Red O Staining

As previously described [17], lipid accumulation was quantified by Oil Red O (ORO) staining. Briefly, HepG2 or differentiated 3T3-L1 cells were fixed with 4% paraformaldehyde (PFA, Electron Microscopy Sciences, Hatfield, PA, USA) and incubated with freshly prepared 0.2 μm-filtered ORO solution (one part of water mixed with three parts of 1 g/100 mL ORO solution in isopropanol) for 30 min. Extensive washing with 60% isopropanol followed by PBS removed the excess stain. Then, photos of cells were taken in order to visualize lipid droplets (not shown). ORO was extracted from the stained cells using 100% isopropanol. The staining was quantified by measuring the optical density at an absorbance of 510 nm.

### 2.13. Interleukin-6 Measurement

As previously described [17], effects of carrot extracts on interleukin-6 production on Raw 264.7 were analyzed by using interleukin-6 ELISA kits (eBioscience).

### 2.14. Statistical Analysis

As previously described [17], for cell experiments (i.e., oxidative stress, proliferation, apoptosis lipid accumulation and il-6 production), values were normalized by the following formula:

X = 100 × [(T − C)/(PC − C)](1)

where T was the value of tested condition, PC was the value of positive control and C was the control in the presence of solvent only. T was the value obtained with carrot of different varieties and storage condition. Positive control was the response obtained with indicated stimuli depending of the functional cell response (i.e., VEGF or FBS for proliferation; actinomycin D for apoptosis; H_2_O_2_ for ROS; dexamethasone/IBMX/insulin or oleic acid for 3T3 cells and HepG2 respectively to quantify lipid accumulation). Data represented the mean of two experiments repeated in triplicate and were expressed as percentage of the positive control. Values exceeding ± 20% of controls were considered biologically significant

Data of carotenoid and polyphenol content were expressed as mean ± SEM. The difference between groups was analyzed by one-way analysis of variance (ANOVA) and post hoc analyses were performed by the multiple-comparison Tukey test. *p* < 0.05 was considered to be statistically significant.

Statistical analyses were performed using R software. Hierarchical clustering was then calculated using the hclust function with “1—Pearson correlation” and “ward.D2” as distance and agglomeration methods respectively. Pearson correlation between cellular results and extract content was computed using R software.

## 3. Results

### 3.1. Carotenoid Content in Carrot Extracts

**Figure 2:**
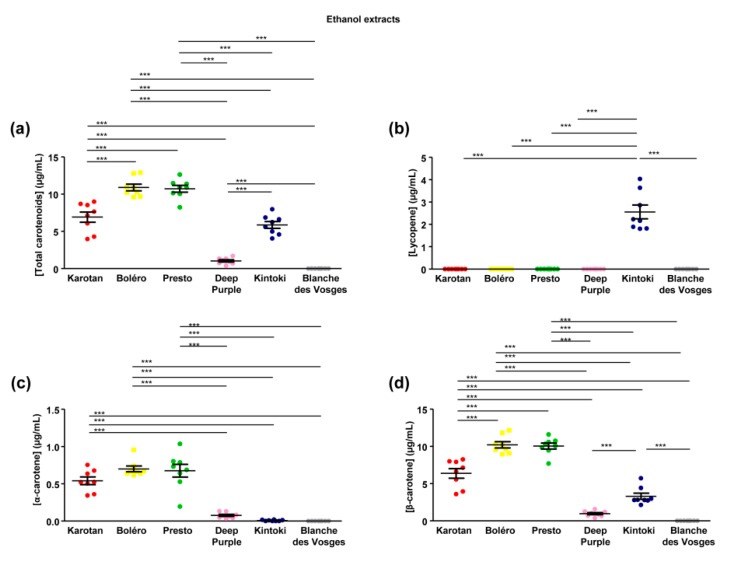
Carotenoid content in carrot’s ethanol extracts. Diagrams represent content of (**a**) total carotenoids, (**b**) lycopene, (**c**), α-carotene, (**d**) β-carotene in ethanol extracts from six different varieties of carrots (Bolero, Presto, Deep Purple, Karotan, Kintoki and Blanche des Vosges), independently from growing conditions. Statistical analyses were performed by one-way ANOVA and Tukey post hoc test, *** *p* < 0.001.

Carotenoid composition of carrot extracts has been analyzed in the ethanol extracts only, since DMSO is a solvent methodologically incompatible with system used in our experimental condition. Carotenoid content ranged between 0 and 10.91 ± 0.45 µg/mL with significant differences depending on the carrot varieties (Figure 2a). Bolero and Presto extracts had the highest content of total carotenoids, followed by Karotan and Kintoki. The lowest content was found in Deep Purple. Carotenoids were absent from the Blanches des Vosges extracts studied.

Three major groups of carotenoids were identified, including lycopene, α-carotene and β-carotene. Lycopene, the precursor of other carotenoids, was detected in Kintoki extract only (Figure 2b). The amount of α-carotene was comparable in Karotan, Bolero and Presto extracts, while it was lowest in Deep Purple and Kintoki (Figure 2c). β-carotene content was the most abundant carotenoids in the five varieties of carrot containing carotenoids and its profile was comparable to the total carotenoid content (Figure 2d).

### 3.2. Polyphenol Content in Carrot Extracts

**Figure 3:**
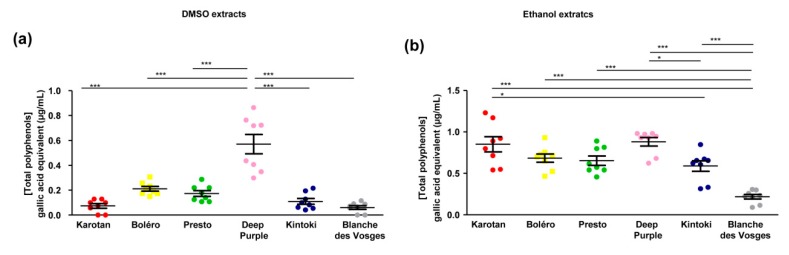
Total polyphenol content in carrot extracts. Diagrams represent content of total polyphenols in DMSO (**a**) and ethanol (**b**) carrot extracts from six different varieties of carrots (Bolero, Presto, Deep Purple, Karotan, Kintoki and Blanche des Vosges), independently from growing conditions and expressed as µg/mL gallic acid equivalents. Statistical analyses were performed by one-way ANOVA and Tukey post hoc test, * *p* < 0.05, *** *p* < 0.001.

Polyphenol content deferred, depending on carrot varieties and the solvent used. In general DMSO extracts contained lower amount of polyphenols compared to ethanol extracts. Indeed, the total polyphenol content in DMSO extracts ranged between 0.061 ± 0.014 and 0.57 ± 0.07 and gallic acid equivalents; while the polyphenol content in ethanol extracts varied between 0.21 ± 0.02 and 0.85 ± 0.09 with significant differences depending on the carrot varieties (Figure 3).

The DMSO extract from Deep Purple had the highest content of total polyphenols compared to other varieties (Figure 3a).

The amount of polyphenols in ethanol extracts of Karotan, Bolero, Presto and Deep Purple was similar. Kintoki extracts contained less polyphenols than Karotan and Deep Purple, while the concentration detected in Blanches des Vosges was the lowest (Figure 3b).

### 3.3. Broad Effect of Carrot Extracts on Vascular, Metabolic and Immune Cell Models

**Figure 4:**
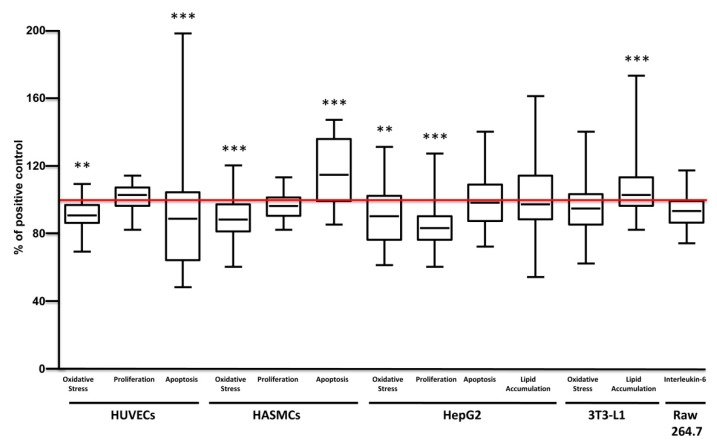
Global bioactivity of carrot extracts on cell models. Each box and whisker plot represent the effect of carrot extracts in the indicated process (Oxidative stress, cell proliferation, cell apoptosis, lipid accumulation, interleukin-6 production) and cell type (HUVECs, HASMCs, HepG2, 3T3-L1, Raw 264.7) independently from solvent, concentration, variety and growing condition. Results were expressed as percentage of positive control (red line). Statistical analyses were performed by one-way ANOVA and Bonferroni post hoc test, ** *p* < 0.01, *** *p* < 0.001 *vs* control.

Broad pharmacological responses of carrot extracts were examined in each cell model independently from solvent, concentration, varieties and growing conditions (Figure 4). Carrot extracts decreased oxidative stress and apoptosis of endothelial cells, without significant effects on proliferation, while in smooth muscle cells they reduced oxidative stress and increased apoptosis without change proliferation.

The oxidative stress observed in endothelial cell was negatively correlated with total polyphenol content (r = −0.342 and *p* = 0.017). Endothelial cell apoptosis was negatively correlated with α-carotene (r = −0.473 and *p* = 0.02). In smooth muscle cells, oxidative stress was positively correlated with α-carotene (r = 0.430 and *p* = 0.030) content, while it was negatively correlated with total polyphenol (r = −0.420 and *p* = 0.003) content.

In hepatocytes, carrot extracts decreased ROS production and cell proliferation with no significant effect on apoptosis and lipid accumulation. In adipocytes, carrot extracts increased lipid accumulation without changing the production of ROS.

In hepatocytes, the effect of proliferation positively correlated with α-carotene (r = 0.663 and *p* < 0.0001), β-carotene (r = 0.569 and *p* = 0.004) and total carotenoid (r = 0.477 and *p* = 0.018), while it was negatively correlated with total polyphenol (r = −0.535 and *p* < 0.0001) content. Whereas, in adipocytes lipid accumulation negatively correlated with α-carotene (r = −0.512 and *p* = 0.011), β-carotene (r = −0.488 and *p* = 0.016), total carotenoid (r = −0.487 and *p* = 0.016) and total polyphenol (r = −0.480 and *p* = 0.001).

Globally, carrots extracts displayed beneficial effects on endothelial and smooth muscle cells, especially in early processes of atherosclerosis, like oxidative stress and apoptosis. Moreover, they reduced oxidative stress and proliferation in hepatocytes and enhanced lipid accumulation in adipocytes. Interestingly, the observed pharmacological responses could be explained by the different extract content as supported by correlations. The detailed effect of carrot extracts on different target cell models considering varieties, growing conditions, extract solvent and concentrations used, and the correlation between the pharmacological effect with extract content, in terms of sugar, carotenoids and polyphenols are described and showed in Appendix A.

### 3.4. Clustering of Cellular Responses to Carrot Extracts

**Figure 5:**
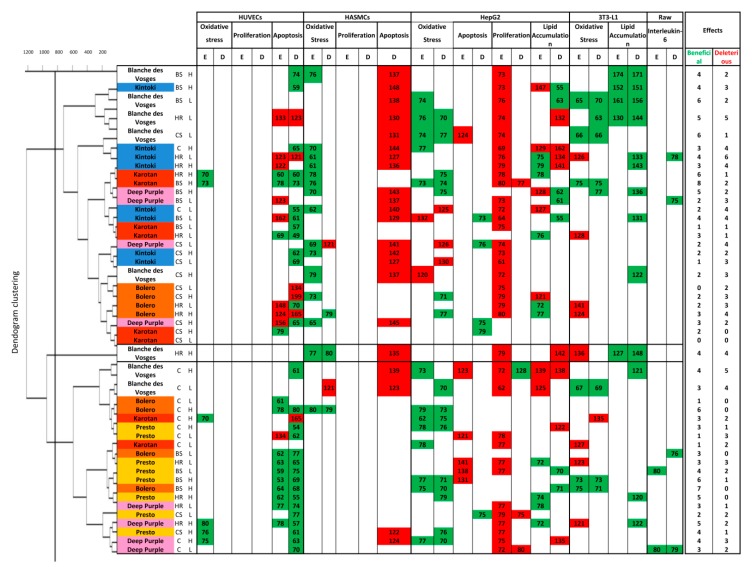
Hierarchical clustering of carrot samples generated from cell pharmacological responses. Cluster dendogram and table showing results obtained in cell models, grouped following clustering. Green cells indicate beneficial effect; red cells correspond to deleterious effect; while white cells show insignificant biological effect.

Hierarchical clustering obtained in function of pharmacological responses revealed three groups of carrot extracts showing different cellular profiles (Figure 5).

In the diagram, green cells indicate beneficial effect; red cell deleterious effect and white cells insignificant biological effect.

Cluster 1 contained cellular responses to six Karotan extracts under BS, HR and CS conditions at the two concentrations used, eight Kintoki extracts independently from condition and concentration, four Bolero extracts under HR and CS conditions at both concentrations, four Deep purple extracts under BS and CS conditions independently from concentrations and five Blanche des Vosges extracts under BS and CS conditions at both concentrations and under HR condition at low concentration only. Samples of cluster 1 reduced endothelial cell apoptosis, oxidative stress in smooth muscle cells, hepatocytes and adipocytes, and facilitated lipid accumulation in adipocytes (Figure 5). They also increased smooth muscle cell apoptosis and reduced hepatocyte proliferation. These results suggest that samples from cluster 1 possess a broad spectrum of target cells, but they were beneficial mostly in adipocytes.

Cluster 2 was composed of only one Blanche des Vosges extract from HR condition at high concentration. This extract decreased ROS production of smooth muscle cells and favored lipid accumulation in adipocytes. However, it increased apoptosis of smooth muscle cells and hepatocytes, increased lipid accumulation in hepatocytes, and oxidative stress in adipocytes, indicating that it was effective on smooth muscle cells and hepatocytes.

Cluster 3 consisted of responses to Presto extracts in any growing conditions, Karotan under C condition, Bolero under C and BS conditions, Deep purple under C and HR conditions and Blanche des Vosges in C condition independently from concentration. Extracts of this cluster decreased apoptosis of endothelial cells and decreased oxidative stress, proliferation and lipid accumulation in hepatocytes. The results indicate that extracts were particularly beneficial in endothelial cells and hepatocytes.

Altogether, the screening indicated that carrot extracts from any varieties were mostly effective and beneficial on endothelial cell apoptosis and on oxidative stress in hepatocytes. Moreover, data revealed that Bolero under BS condition at high concentration was the most efficient with 7 beneficial effects without any deleterious ones.

### 3.5. Ranking of Effects

**Figure 6:**
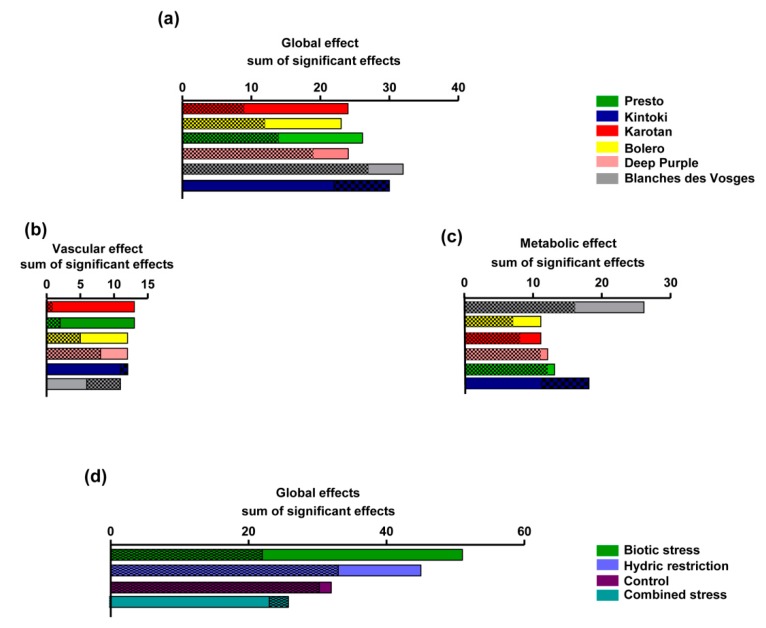
Ranking of the pharmacological effects generated from the cellular responses. Sum of the pharmacological responses that were significantly beneficial (not dotted histograms) or deleterious (dotted histograms) on all examined cells (**a**), with respect to varieties on endothelial and smooth muscle cells (**b**) and hepatocytes and adipocytes (**c**). Sum of the significantly different pharmacological responses that were beneficial and deleterious observed in all types of cells considering different storage growing conditions (**d**). Beneficial effects are represented by no-dotted histograms while deleterious effects by dotted histograms.

Carrot extracts exhibited more significant beneficial effects (58%) than deleterious effects (42%) under the experimental conditions performed in the present study. The pharmacological responses of carrot varieties were summarized in Figure 6a. Karotan had the best beneficial/deleterious effect ratio (2.7), followed by Bolero (1.9), Presto (1.8), Deep Purple (1.3), Blanche des Vosges (1.18) and Kintoki (0.7). Regarding vascular cells (Figure 6b), Karotan had the best beneficial/deleterious effect ratio (13), followed by Presto (6.4), Bolero (2.4), Deep Purple (1.5), Kintoki (0.9) and Blanche des Vosges (0.5). Looking at metabolic cells (hepatocytes and adipocytes) (Figure 6c) Blanche des Vosges had the best beneficial/deleterious effect ratio (1.65), followed by Bolero (1.6), Karotan (1.4), Presto and Deep Purple (1.1), Kintoki (0.6). This evaluation showed that Karotan was the most efficient varieties on endothelial and smooth muscle cells, while Blanche des Vosges was the most effective on hepatocyte and adipocytes.

The sum of the significant effects of growing conditions on all types of cells (Figure 6d) indicated that biotic stress conditions have the best ranking of beneficial effects followed by hydric restriction, control and combined stress conditions. Indeed biotic stress represented the growing condition having 34% of beneficial effects, followed by hydric stress (30%), controlled (21%) and combined stress (15%). Therefore, biotic stress was the most beneficial carrot growing condition on examined cells.

## 4. Discussion

The present report allowed the identification of the most effective carrot extract by concomitant evaluation of multiple parameters, including variety, growing condition, solvent and concentration. Additionally, it evidenced the beneficial effects of carrot extract on endothelial cells by decreasing oxidative stress and apoptosis and on smooth muscle and hepatocytes by decreasing oxidative stress; while revealed contrasted effect on smooth muscle cell apoptosis and hepatocyte proliferation. Among varieties, Karotan and Presto exhibited endothelial tropism, while Blanche des Vosges displayed adipocyte tropism. Interestingly, extracts from carrot in biotic stress condition were the most beneficial, while those from combined stress were the least effective.

Although observed pharmacological responses have been correlated to carotenoids and polyphenol content, other nutritional components including sugar, fibers and polyacetylene could be play a role in the healthy properties of carrot extracts [21,22].

In present study, six genotypes contrasted by their root color have been considered. It is well established that orange carrot genotypes contain mostly α-carotene and β-carotene, yellow genotypes contain lutein, the red ones contain lycopene, and white genotypes contain almost no carotenoids [23]. Among carrots with the same color, total carotenoid content can vary greatly [6,24]. In line with these evidences, we found that Deep Purple and Blanche des Vosges extracts had less carotenoid content and Kintoki extract only contained lycopene. Moreover, polyphenol content in ethanol extracts was greater than those of DMSO extracts, except for Deep Purple. This observation is in line with the fact that ethanol is a better solvent for polyphenols extraction [25].

From all the pharmacological responses studied, carrot extracts displayed beneficial properties mostly by their capacity to reduce oxidative stress in endothelial and smooth muscle cell and in hepatocytes. Interestingly, vascular oxidative stress negatively correlated with total polyphenol content. Vascular protection could be associated with the potential radical scavenging activity of pigment-rich fruits and vegetables, including carrot, that lead to increased nitric oxide bioavailability, decreased endothelial apoptosis and hence increased vasodilatation [26]. These effects might be related to the antioxidant properties of either carotenoids or polyphenols or both [27].

Regarding the hepatocyte function, the ability of carrot extracts to reduce proliferation might be involved in their beneficial property and allow a significant protective action in the alleviation of liver injury [8]. A large number of secondary plant substances, including carotenoids and polyphenols, reduce cellular necrosis and proliferation, key processes during carcinogenesis [28]. Dietary carotenoid-rich extracts of carrots inhibit biochemical and cellular events, including proliferation, that play a role in the early stages of hepatocarcinogenesis [28]. The present study highlights the role of polyphenol contents demonstrating an inverse correlation between polyphenols content and their ability to reduce hepatocyte proliferation.

Concerning the adipocytes function, carrot extracts increased lipid accumulation. This effect was negatively correlated with α-carotene, β-carotene, total carotenoid, and total polyphenol contents and hence might participate in the reduction of adiposity and improve adipose tissue function. These results are in line with those reported in the literature showing that the beneficial effects of carrot on adipose tissue can be attributed to their carotenoid and polyphenol content [29,30]. Moreover, among other components present in carrots, sugar may interfere with several adipocyte functions as both fructose and sucrose are recognized to induce adipogenesis [31,32]. Sugar content of the carrot might participate to the reduced fat accumulation observed in the present study. Further work is needed to analyze further the relative contributions of fructose and sucrose in these effects.

The pharmacological strategy used discriminated the properties of carrot extracts by taking in consideration various parameters, including different carrot varieties and growing conditions. The clustering study revealed three groups. The first cluster displayed beneficial property mostly on adipocytes function; the second cluster (made of high concentration of Blanche des Vosges extract under HR condition only), on smooth muscle cells and hepatocytes; and the third cluster on endothelial cells and hepatocytes. Further analyses led allowed us to identify the vascular and metabolic profiles of each carrot variety. Karotan and Presto extracts exhibited endothelial tropism while Blanche des Vosges extracts had an adipocyte tropism. Differences in the polyphenol and carotenoid contents in each variety could explain, at least in part, the differential pharmacological responses observed. However, we cannot exclude the contribution of other carrot components such as polyacetylenes, a large group of nonvolatile phytochemicals, exhibit potent anti-inflammatory and anticancer effects that might also contribute to the health benefits associated with carrot consumption [22,33,34].

We also found that growing conditions impact the effect of carrot extracts. Among the examined growing conditions, extracts from carrot grew under biotic stress displayed the most beneficial effects on any cell type studied, while the combined stress condition was the least effective. In carrot roots, the biotic and combined stress conditions globally led to a decrease in carotenoid contents whatever the genotype. Conversely, the effect of water restriction condition on carotenoid accumulation was dependent on the genotype [18]. Despite abiotic and biotic stresses applied separately or in combination may have an impact on the overall carrot root composition, we did not observe any significant modification in its polyphenol and carotenoid contents under these different growing conditions (data not shown).

Finally, the approach used highlighted that the extract of Bolero from biotic stress condition at high concentration was the most effective under the experimental conditions used. Another study evidenced the healthy effect of Bolero carrots. Indeed, root Bolero carrots stimulate immune response on both intestinal and peripheral immunity in mice, including an expansion of Treg cells [35]. Moreover, salicylic acid content in root Bolero carrot has been shown to be significantly increased under *A. dauci* inoculation [17]. Salicylic acid is a hormonal mediator of the systemic acquired resistance response to pathogen attack and environmental stress [36] and could represent another possible candidate contributing to the beneficial properties of carrot root extract.

### Limitations of the Study

Apparent discrepancies in the present study could be due to i) the evaluation of concomitant but independent cellular process in isolated in vitro models; and ii) the possible interaction between compounds. In fact, the present study does not take in consideration the bioavailability, metabolism and interaction of active compounds. For example, some in vivo studies have shown a competition of carotenoids to be micellized or absorbed. There are contrasted evidences about a decrease of lutein when it is consumed along with lycopene or β-carotene and of α-carotene and lycopene after β-carotene uptake, [37]. As consequence, the evaluation of the effects of carrot using in vivo models is mandatory to further increase our knowledge. Finally, this report particularly emphasized the relation between the protective effects of carrot extract and their content in carotenoid and polyphenol. However, the observed health properties of extracts could also be attributed to their sugar, fiber and/or polyacetylene content and a more in-depth consideration of all compounds present in those extracts is advisable. Indeed, the identification of phytochemical family and/or compound that exhibited a pharmacological profile comparable to the original extract would let to examine and precisely decipher the mechanisms of action of extracts, which was a limitation of the present study.

## 5. Conclusions

The multi-factorial strategy elaborated for this study allowed a screening on cell models related to vascular and metabolic dysfunctions including endothelial and smooth muscle cells, hepatocytes, adipocytes and macrophages. It also allowed the evaluation of pharmacological response efficiency relation to carrot polyphenol and carotenoid contents. This approach also confirmed that carrot extracts represent a possible dietary source of beneficial natural compounds for human nutrition and health. For instance, the Bolero extract from biotic stress condition at high concentration could be a good candidate for the formulation of effective nutraceuticals to improve endothelial, hepatic and adipocyte functions. Finally, it reinforced the knowledge of the beneficial nutraceutical properties of carrot roots and represented a source for the use of certain extracts as effective dietary supplements associated with the treatment and/or the management of vascular and metabolic disorders.

## Figures and Tables

**Figure A1:**
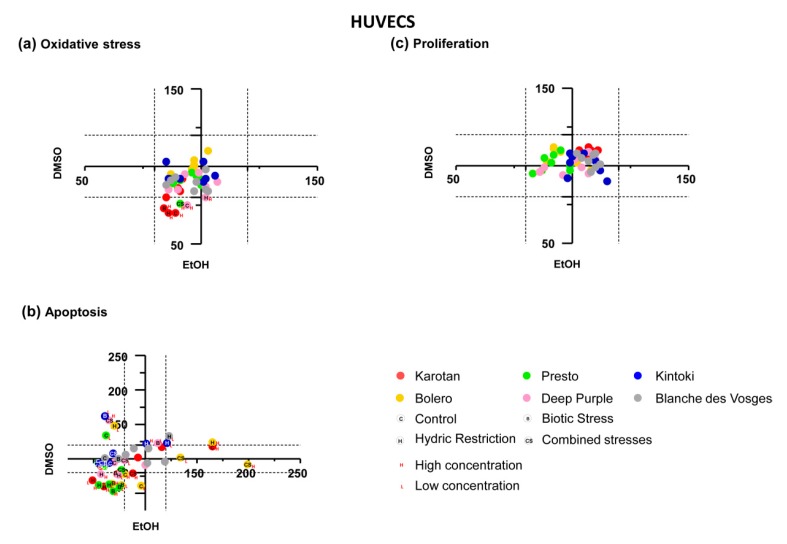
Effect of carrot extracts on endothelial cells. Oxidative stress (**a**), apoptosis (**b**) and proliferation (**c**) in HUVECs after treatment with carrot extract from different carrot varieties and growing conditions. In each diagram, the x-axis and y-axis expressed percentage response values of ethanol and DMSO samples, respectively. Variations of less than 20% of responses (included in dot lines) were not considered biologically significant.

**Figure A2:**
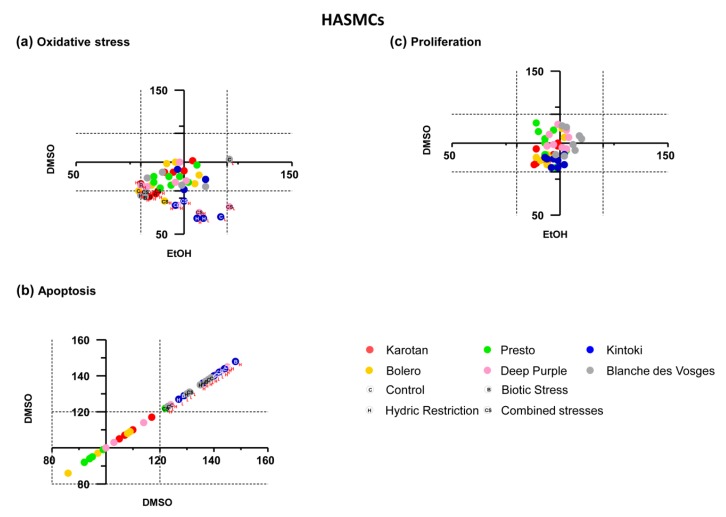
Effect of carrot extracts on smooth muscle cells. Oxidative stress (**a**), apoptosis (**b**) and proliferation (**c**) in HASMCs after treatment with carrot extract from different carrot varieties and growing conditions. In each diagram, x-axis and y-axis expressed percentage response values of ethanol and DMSO samples, respectively. Variations of less than 20% of responses (included in dot lines) were not considered biologically significant.

**Figure A3:**
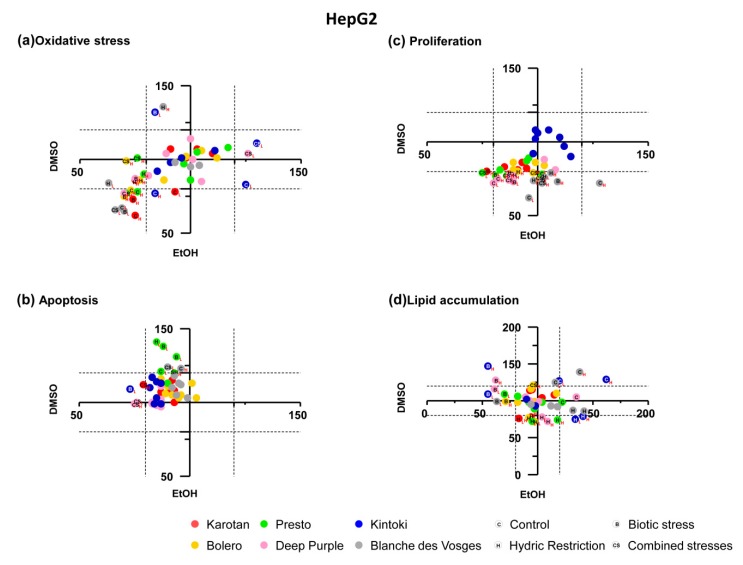
Effect of carrot extracts on hepatocytes. Oxidative stress (**a**), apoptosis (**b**), proliferation (**c**) and lipid accumulation (**d**) in HepG2 after treatment with carrot extract from different carrot varieties and growing conditions. In each diagram, x-axis and y-axis expressed percentage response values of ethanol and DMSO samples, respectively. Variations of less than 20% of responses (included in dot lines) were not considered biologically significant.

**Figure A4:**
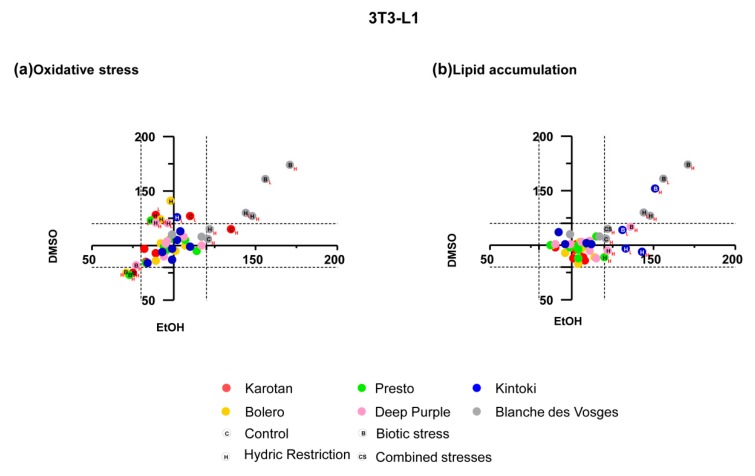
Effect of carrot extracts on adipocytes. Oxidative stress (**a**) and lipid accumulation (**b**) in 3T3-L1 after treatment with carrot extract from different carrot varieties and growing conditions. In each diagram, x-axis and y-axis express percentage response values of ethanol and DMSO extracts, respectively. Variations of less than 20% of responses (included in dot lines) were not considered biologically significant.

**Figure A5:**
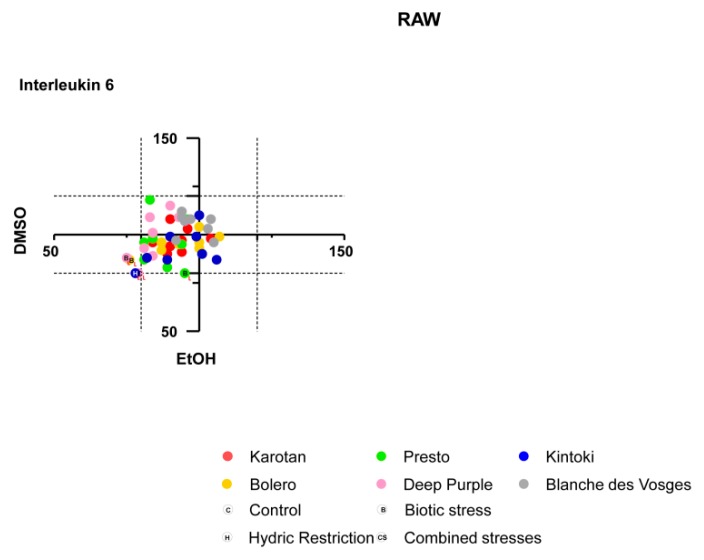
Effect of carrot extracts on macrophages. IL-6 production in Raw 264.7 after treatment with carrot extract from different carrot varieties and growing conditions. In diagram, x-axis and y-axis expressed percentage response values of ethanol and DMSO extracts, respectively. Variations of less than 20% of responses (included in dot lines) were not considered biologically significant.

## Appendix A

### Appendix A.1. Effect on Endothelial Cells

The ability of carrot root extracts to prevent oxidative stress and apoptosis, and to enhance proliferation in endothelial cells was considered beneficial.

As shown on Figure A1a, Karotan extracts under C, BS and HR conditions, Presto under CS condition and Deep Purple under C and HR conditions decreased ROS production at high concentration in ethanol only.

Karotan extracts, under BS condition at high concentration in both solvents; under HR condition at the two concentrations and solvents used; and under CS condition at high concentration in ethanol only, decreased endothelial cell apoptosis (Figure A1b). Whereas Karotan under C condition, at high concentration and in DMSO only increased cell death. Presto under C condition at high concentration in DMSO and at low concentration in ethanol decreased apoptosis, while at high concentration in DMSO increased this process. Presto under HR and BS conditions independently from concentration and solvent and under CS condition at low concentration and in DMSO decreased cell death. Kintoki under C and BS conditions at both concentrations and in DMSO only reduced apoptosis. Whereas Kintoki under BS condition at low concentration and in ethanol, under HR condition at two concentrations used in ethanol and at low concentration DMSO increased apoptosis. Bolero under C condition at two concentrations used in ethanol and at high concentration in DMSO reduced cell death. The same effect was observed by testing Bolero under BS condition independently from concentration and solvent. Deep Purple under C condition at both concentrations in DMSO, under HR condition at both concentrations and solvents and under CS condition at high concentration in DMSO decreased apoptosis, while Deep purple, under CS condition at high concentration, increased endothelial cell death. Finally, Blanche des Vosges under BS condition at high concentration in DMSO reduced apoptosis, whereas under HR conditions at low concentration in both solvents enhanced apoptosis.

Surprisingly, endothelial apoptosis negatively correlated with β-carotene (r = −0.410 and *p* = 0.047) content of ethanol extracts at high concentration, and α-carotene (r = −0.439 and *p* = 0.032; r = −0.418 and *p* = 0.032 for high and low concentration respectively) content of ethanol extracts, whatever the concentration used.

Among extracts, Karotan in BS and HR conditions and Deep purple under HR condition at high concentration were the most effective on endothelial cell functions such as reduction of oxidative stress and apoptosis. Some of the observed effects were associated to differential carotenoid content of extracts as suggested by significant correlations.

However, endothelial cell proliferation was not modified by any extract whatever the variety, growing condition, concentration or solvent used (Figure A1c).

### Appendix A.2. Effect on Smooth Muscle Cells

The ability of extracts to decrease oxidative stress, apoptosis and proliferation in smooth muscle cells was considered beneficial. Figure A2a showed that Karotan under BS and HR conditions at high concentration and in ethanol only decreased ROS production in smooth muscle cells. Kintoki under C and HR conditions at the two concentrations, and under CS condition at high concentration in ethanol only, reduced oxidative stress. Bolero under C condition at high concentration in both solvents, and under HR conditions at high concentration in DMSO only, decreased ROS level. Deep Purple under BS condition at high concentration, and under CS condition at low concentration in ethanol reduced oxidative stress. In contrast, Deep Purple under CS condition at low concentration in DMSO increased ROS production. Blanche des Vosges under BS and HR conditions at high concentrations in ethanol decreased oxidative stress. Whereas, Blanche des Vosges under C condition at low concentration in DMSO increased ROS level.

Interestingly, oxidative stress in smooth muscle cells positively correlated with α-carotene (r = 0.417 and *p* = 0.043) content of ethanol extracts at low concentration.

Apoptosis of HASMCs has been evaluated using carrot extracts solubilized in DMSO since EtOH decreased apoptosis by itself under the experimental conditions used (data not shown). Kintoki under C, HR and CS at both concentrations increased smooth muscle cell apoptosis (Figure A2b). Deep Purple under C condition at high concentration, under BS and CS conditions at both concentrations enhanced apoptosis. Blanche des Vosges under C condition at both concentrations, under BS and HR conditions at high concentration and under CS condition at low concentration displayed a pro-apoptotic effect.

Smooth muscle cell proliferation was not modified by any extract whatever the variety, growing condition, concentration or solvent used (Figure A2c).

In summary, carrot extracts were effective in reducing oxidative stress in smooth muscle cells. This effect could be partly linked to their carotenoid contents.

### Appendix A.3. Effect on Hepatocytes

The capacity of extracts to prevent oxidative stress, apoptosis and lipid accumulation as well as their ability to increased proliferation of hepatocytes was considered beneficial.

As shown in Figure A3a, Karotan reduced oxidative stress under C condition in ethanol only at low concentration and at high concentration in both solvent, under BS condition in both solvents and under HR condition in DMSO only at high concentrations. Presto under C and BS conditions in both solvents and under HR and CS condition in DMSO only at high concentrations decreased ROS production. Kintoki under C condition prevented oxidative stress at high concentration in ethanol only, while under C condition in DMSO, under BS condition in ethanol and under CS condition in DMSO at low concentration increased ROS levels. Bolero under C and BS conditions in both solvents and under HR and CS conditions in DMSO only at high concentration reduced oxidative stress. Deep Purple under C condition in both solvents and under BS condition in DMSO only at high concentration reduced ROS. In contrast, Deep Purple under CS in DMSO only at low concentration increased oxidative stress. Finally, Blanches des Vosges under C condition in DMSO at low concentration, in ethanol at high concentration, under BS condition at low concentration in ethanol, under HR and CS conditions in both solvents at low concentrations reduced oxidative stress. However, Blanches des Vosges under CS condition in ethanol at high concentration increased ROS production.

Karotan extracts reduced HepG2 apoptosis under CS condition at high concentration in ethanol only. Presto under C condition at low concentration, under BS condition at both concentration and under HR condition at low concentration in ethanol increased apoptosis, while under CS condition at low concentration in DMSO only it reduced this process (Figure A3b). Kintoki under BS condition at low concentration and Deep Purple under CS condition at both concentrations in DMSO decreased apoptosis. In contrast, Blanche des Vosges under C condition at high concentration and under CS condition at low concentration in ethanol increased cell death.

Karotan under HR condition at both concentrations reduced lipid accumulation in hepatocytes in ethanol only (Figure A3d).

Proliferation was reduced by Karotan under C and BS conditions at low concentration in ethanol only, also under BS condition at high concentration in both solvents and under HR condition at high concentration in ethanol only (Figure A3c). Additionally, Presto under C, BS and HR conditions at low concentration in ethanol only, under CS condition at low concentration in both solvent and at high concentration in ethanol only; Kintoki independently of grow condition and concentration reduced proliferation in ethanol only. Bolero under HR and CS at both concentration and in ethanol only and Deep Purple under C condition at low concentration in both solvent, at high concentration in ethanol only, under BS condition at low concentration in ethanol only, under HR condition at both concentration and under CS condition at low concentration in ethanol only, decreased proliferation. Blanches des Vosges under C condition at high concentration in DMSO only increased proliferation, while under other conditions independently from concentration decreased proliferation in ethanol only. Finally, proliferation of hepatocytes positively correlated with β-carotene (r = 0.519 and *p* = 0.024) and total carotenoids (r = 0.435 and *p* = 0.036) of ethanol extracts at low concentration. Presto under C condition at high concentration in DMSO only increased lipid accumulation, whereas it decreased lipid accumulation under BS condition at low concentration in DMSO and under HR condition at both concentrations in ethanol (Figure A3d). Kintoki under C condition at both concentrations in ethanol and at high concentration in DMSO increased lipid accumulation. Under BS condition at high concentration in ethanol only also favored lipid accumulation, while in DMSO at both concentrations decreased it. Under HR condition, Kintoki extracts decreased lipid accumulation in ethanol, but increased it in DMSO independently from concentration. Bolero under BS condition at high concentration in DMSO, under HR condition at both concentrations in ethanol reduced lipid accumulation. To the contrary, under CS condition at high concentration in ethanol only, it enhanced it. Deep purple under C condition in DMSO and under BS condition in ethanol increased lipid accumulation at high concentration. In contrast, under BS condition at both concentrations in DMSO and under HR condition independently from concentration in ethanol only it decreased lipid accumulation. Finally, Blanche des Vosges under BS condition at low concentration in DMSO decreased lipid accumulation, whereas, under C condition at low concentration in ethanol, at high concentration independently from solvent and under HR condition at both concentrations in DMSO only increased lipid accumulation.

Most of ethanol extracts decreased proliferation. Among extracts, Bolero under BS condition at high concentration was the most effective on hepatic functions such as reduction of oxidative stress and lipid accumulation.

### Appendix A.4. Effect on Adipocytes

The capacity of extracts to prevent oxidative stress and increased lipid accumulation in adipocytes was considered beneficial.

Karotan under C and HR conditions at low concentration in ethanol and under C condition at high concentration in DMSO only increased oxidative stress (Figure A4a). In contrast under BS condition at high concentration independently from solvent Karotan decreased ROS. Presto under BS condition at high concentration in both solvents reduced ROS production, whereas under HR condition at low concentration in ethanol only increased it. Kintoki under HR condition at low concentration in ethanol only enhanced ROS level. Bolero under BS condition at high concentration in the two solvents decreased oxidative stress, while under HR condition at the two concentrations in ethanol only increased ROS. Deep purple under BS condition at high concentration in DMSO only decreased ROS production, whereas under HR condition at high concentration in ethanol only increased oxidative stress. Blanche des Vosges under C, BS and CS conditions at low concentration in both solvents, and under HR condition at low concentration in DMSO only decreased oxidative stress, while Blanche des Vosges increased ROS under HR condition at high concentration in ethanol only. Curiously, oxidative stress in adipocyte positively correlated with β-carotene (r = 0.420 and *p* = 0.041), total carotenoid (r = 0.456 and *p* = 0.025) and total polyphenol (0.567 and *p* = 0.004) content of ethanol extracts at low concentration.

With regards to lipid accumulation, Presto under HR condition at high concentration in DMSO only favored this process (Figure A4b). Also Kintoki under BS condition at low concentration in DMSO, at high concentration in both solvent; under HR condition at the two concentrations used in DMSO only increased lipid accumulation. The same effect occurred with Deep Purple under BS and HS conditions at high concentration in DMSO only. Similarly, Blanche des Vosges under C condition at high concentration in DMSO only, under BS and HR conditions independently from concentration and solvent and under CS condition at high concentration in DMSO only favored lipid accumulation. Interestingly, lipid accumulation negatively correlated with α-carotene (r = −0.483 and p = 0.017; r = −0.513 and *p* = 0.010 for high and low concentration respectively), β-carotene (r = −0.441 and *p* = 0.031; r = 0.515 and *p* = 0.010 for high and low concentration respectively), total carotenoid (r = −0.434 and *p* = 0.034; r = −0.523 and *p* = 0.009 for high and low concentration respectively) and total polyphenol (r = −0.548 and *p* = 0.006; r = −0.612 and *p* = 0.001 for high and low concentration respectively) content of ethanol extracts independently from concentration.

Among extracts, Blanche des Vosges under BS condition at low concentration was the most effective on adipocytes capacity to reduce oxidative stress and increase lipid accumulation concomitantly.

### Appendix A.5. Effect on Macrophages

The ability of extracts to decrease pro-inflammatory cytokine IL-6 in macrophages was considered beneficial.

Presto under BS condition in ethanol, Kintoki under HR condition in DMSO, Bolero under BS condition in DMSO, and Deep Purple under C condition in the two solvents and under BS in DMSO at low concentration reduced IL-6 production (Figure A5).
